# The German simpler modified fried frailty scale: translation, cross-cultural adaptation and clinical validation

**DOI:** 10.1186/s12877-026-07134-1

**Published:** 2026-03-05

**Authors:** Sabine Schluessel, Gulistan Bahat, Linda Deissler, Christopher Held, John Michael Hoppe, Sebastian Martini, Katharina Mueller, Michaela Rippl, Ralf Schmidmaier, Olivia Tausendfreund, Michael Drey

**Affiliations:** 1 Department of Medicine IV, LMU University Hospital, LMU Munich, Munich, Germany; 2 Istanbul Medical Faculty, Department of Internal Medicine, Division of Geriatrics, Istanbul University, Istanbul, Turkey

**Keywords:** Frailty, Practical, Screening, Fried, Validation

## Abstract

The Simpler Modified Fried Frailty Scale (SFS) was developed for the rapid evaluation of physical frailty in older adults. The aim of this study was to translate, culturally adapt and validate the German version of the SFS using the Fried Frailty Phenotype (FFP) as a reference. The methodological implementation was carried out in two phases: First, the German version was translated from the original English version according to established standards, culturally adapted and tested for comprehensibility in a small sample. Interrater reliability and test-retest reliability were assessed in 20 older patients. This was followed by clinical validation in a sample of 100 geriatric participants (mean age 82 ± 6.5 years; 66% women). The German SFS proved to be understandable and reliable. Internal consistency was moderate with a Cronbach’s alpha of 0.692. Agreement with the FFP was substantial (Cohen’s kappa = 0.80; *p* < 0.001). All five individual items showed significant positive correlations with the total score and with the corresponding FFP domains. In regression analyses, the total SFS score was significantly associated with frailty status (OR = 2.954; 95% CI: 1.877–4.649; *p* < 0.001), with unintentional weight loss, fatigue and physical inactivity showing particularly high discriminatory power. A cut-off value of ≥ 3 points showed a sensitivity of 91.67%, a specificity of 88.46%, a positive predictive value of 88.00% and a negative predictive value of 92.00%. The likelihood ratios (LR) were LR⁺ = 7.97 and LR⁻ = 0.09. The ROC analysis yielded an area under the curve (AUC) of 0.817 (95% CI: 0.734–0.899), confirming the good diagnostic accuracy of the scale. Overall, the German SFS proved to be a practical, reliable and valid instrument for identifying frailty in older people and is suitable for use in research and everyday clinical practice.

**Supplementary Information:**

The online version contains supplementary material available at 10.1186/s12877-026-07134-1.

## Introduction

### Screening in the age of efficiency

 As societies continue to age, systematic screening for physical frailty is emerging as a health policy priority [1, 2]. Frailty is common among older adults, with prevalence estimates in community-dwelling populations ranging from approximately 7–15%, while an additional 35–45% are considered pre-frail, depending on age, setting, and assessment method [3, 4]. The identification of vulnerable older people enables early preventive measures – and thus the preservation of autonomy, mobility and quality of life [5]. Moreover, detecting frailty is also essential for managing common chronic conditions in older adults such as hypertension, diabetes, cancer, osteoporosis, or sarcopenia, as treatment strategies must be adapted to the individual’s frailty status [6–8]. At the same time, the healthcare system is under pressure to implement diagnostics quickly, easily and in a resource-saving manner. Classic instruments for measuring frailty, such as the Fried Frailty Phenotype (FFP), are hardly practical in many clinical contexts due to the time and equipment they require [9, 10]. Therefore, screening methods are needed that strike a balance between diagnostic accuracy and pragmatic applicability – especially in the daily work of general practitioners and inpatients [9].

### From the fried frailty phenotype to the simpler modified fried frailty scale

The FFP, developed by Fried et al. in 2001, is considered one of the most frequently used scales for phenotypic assessment of physical frailty [4]. It is based on five central dimensions: unintentional weight loss, exhaustion, muscle weakness (measured by handgrip strength), reduced walking speed (measured over a distance of 4.57 m), and low physical activity (assessed using the Minnesota Leisure Time Physical Activity Questionnaire) [4]. If three or more of these criteria are present, the person is defined as frail [4]. In numerous studies, the scale has demonstrated a high prognostic validity with regard to mobility loss, falls, hospitalization and mortality [11–13].

Despite this relevance, the application of the FFP in everyday clinical practice is often limited. In particular, objective measurements – such as handgrip strength via dynamometer, gait speed over defined walking distances and physical activity based on elaborate calorie calculations – are time-consuming, resource-dependent and require a certain degree of cognitive patient cooperation. This is a significant hurdle, especially in geriatric care, which is often characterized by limited time and multiple restrictions of those affected [14, 15].

To address these challenges, the Simpler Modified Fried Frailty Scale (SFS) was developed by Bahat et al. as a practical alternative [16]. While remaining conceptually aligned with Fried’s original model, the SFS operationalizes the five core criteria in a simplified and more feasible format. Two dimensions – unintentional weight loss and exhaustion/fatigue – remain unchanged [16]. The other three dimensions, however, have been specifically modified to ease the diagnostic process and broaden applicability in routine care settings [16].

In the SFS, objective physical performance measures such as handgrip strength, gait speed, and physical activity levels have been replaced with standardized questions [16]. These are designed to elicit subjective assessments of frailty-related domains and can be answered either by the individual themselves or, if needed, by caregivers or nursing staff [16]. Specifically, muscle weakness is assessed through a question comparing perceived handgrip strength to that of healthy peers of the same age [16]. Walking speed is similarly evaluated through self- or proxy-assessment by comparing the individual’s gait to that of age-matched, healthy individuals [16]. Physical activity is no longer quantified via caloric expenditure, but rather assessed through a question asking whether the individual’s activity level over the past week was reduced compared to that of age-matched, healthy individuals [16].

These three simplified components enable a significantly more flexible, resource-efficient, and broadly applicable assessment of frailty – both in general practice, care facilities, and inpatient settings [16]. At the same time, initial studies show that despite its simplification, the SFS is comparably predictive of mortality as the more complex original model [9, 17]. Compared with the original FFP, the SFS is more feasible for individuals with cognitive impairment, as it relies on simple standardized questions rather than performance-based assessments and allows for caregiver or proxy input [16]. In summary, the SFS is a promising instrument that retains the original concept of Fried but is more suited to clinical practice.

### Opportunities of a validated instrument and aim of the study

The use of the SFS promises to significantly simplify frailty screening in clinical and primary care settings for older adults. However, a standardized and scientifically grounded approach to translation and cultural adaptation and validation is essential for the effective implementation of such tools across different linguistic, cultural and clinical contexts. In the case of the SFS, this prerequisite has already been met through a methodologically rigorous report, which ensures a valid and culturally sensitive foundation for its application in German-speaking healthcare settings [16]. So far, there is no German version of the SFS, although similar developments have already been initiated in other European countries like Poland and Spain.

In summary, the aim of the present study was to systematically translate, culturally adapt and psychometrically validate the German version of the SFS according to the internationally recognized methodological protocol of Bahat et al. [16].

## Methods

The validation of the German version of the SFS was carried out in a two-phase study design based on the international consensus protocol [16]:

### Phase 1: translation, cultural adaptation and reliability testing

The first phase involved the systematic translation and cultural adaptation of the scale, followed by a reliability test. The translation was initially carried out by a bilingual geriatrician with German as her first language (Step 1). Subsequently, this first version was reviewed for conceptual accuracy and linguistic comprehensibility together with two other bilingual experts, one of whom had already been involved in validation projects (Step 2). The resulting discussion led to a consensus-based German version.

To ensure accuracy of content, a back-translation was then performed by an independent English native speaker who was not familiar with the study design (Step 3). A panel of experts consisting of the translators and two geriatric specialists compared the back-translation with the original English text and agreed on a final version (Step 4). This was then sent to the first author of the original scale (Gulistan Bahat) and an English-speaking geriatric expert for review and approval (Step 5).

The comprehensibility and cultural appropriateness of the final version were examined in a pretest with ten older people (≥ 65 years, without relevant cognitive limitations or acute functional impairments) (Step 6). Subsequently, the interrater reliability of the scale was assessed in a sample of 20 participants, with two independent geriatricians administering the scale separately (Step 7). Two weeks later, the same subjects were reassessed by one of the two examiners to evaluate the test-retest reliability (Step 8). The intraclass correlation coefficient (ICC) and their 95% confidence intervals (CIs) were calculated using a two-way mixed-effects model with absolute agreement. According to the protocol, reliability was defined as follows: ICC estimate ≥ 0.90: excellent reliability, between 0.75 and 0.9: good reliability, 0.5–0.75: moderate reliability, < 0.5: poor reliability [15]. P-value < 0.05 is considered as statistically significant.

### Phase 2: clinical validation

In the second phase, the clinical validity of the German SFS was evaluated against the reference standard FFP. For this purpose, older adults aged 65 and older who lived independently, had sufficient cognitive abilities (MMSE ≥ 24) and were able to walk at least 6 m without assistance, walking aids permitted, were recruited. Individuals with illnesses that could significantly impair a standardized handgrip strength measurement, such as severe osteoarthritis or neurological limitations, were excluded. According to the international consensus paper specifically addressing the validation of the SFS, which recommends a sample size of 50–100 participants, we selected the upper limit of this range [16]. Between March 2022 and March 2023, 100 participants were recruited from inpatient and outpatient facilities of the Department of Geriatrics at the Ludwig-Maximilian-University (LMU) hospital in Munich, Germany. All participants provided written informed consent prior to enrollment. The Ethics Committee of the Medical Faculty of the LMU approved the study (study no. 23–0434).

In addition to collecting basic data such as age, sex, height, weight, education, and living situation, information on chronic illnesses, medication use, smoking and alcohol consumption, falls, functional status, nutritional status, physical performance, and muscle strength was also documented and is described in the following paragraph (“Measurements”). Following the approach used in the original FFP, patients reaching a score of three or more points were classified as frail. Baseline characteristics were compared between frail patients and controls using either the t-test for continuous variables or the chi-square test for categorical variables, as appropriate.

To assess the discriminative power of the SFS total score and its individual components in identifying frailty, binary logistic regression analyses were performed using FFP as the reference classification (frail: FFP ≥ 3; control: FFP < 3). Each SFS item, as well as the total score, was entered as an independent variable in separate models. All analyses were adjusted for age and sex to control for potential confounding.

The internal consistency of SFS was calculated using Cronbach’s alpha. Construct validity of the German version of the SFS was examined through its correlation with the original FFP. Agreement between the two instruments was evaluated using Cohen’s kappa, interpreted as follows: values between 0.01 and 0.20 indicate slight agreement, 0.21 to 0.40 fair, 0.41 to 0.60 moderate, 0.61 to 0.80 substantial, and 0.81 to 1.00 almost perfect agreement. Diagnostic accuracy was further evaluated by calculating sensitivity, specificity, positive and negative predictive values (PPV and NPV), as well as positive and negative likelihood ratios (LR⁺ and LR⁻). Additionally, a receiver operating characteristic (ROC) curve analysis was performed, using FFP as the reference standard (frail vs. non-frail). The area under the curve (AUC) was calculated to assess overall test performance, with values interpreted as follows: 0.5 no discrimination, 0.7–0.8 acceptable, 0.8–0.9 good, and > 0.9 excellent.

IBM^®^ SPSS^®^ Statistics Version 29 was used for the statistical analyses. Metric variables were presented as mean values with standard deviations (SD), categorical variables as absolute and relative frequencies. Statistical significance was assumed at a p-value < 0.05.

### Measurements

Height (cm) and weight (kg) were measured. Handgrip strength (kg) was assessed using a calibrated hydraulic dynamometer (Jamar, Los Angeles, CA) with the participant seated, the elbow flexed at 90°, and the wrist in a neutral position. Each hand was measured three times, and the maximum value was recorded. Gait speed at a normal pace was measured over a distance of 4 m from a standing position, and the 4.57-meter gait speed test was subsequently calculated. Low physical activity was evaluated using the Minnesota Leisure Time Physical Activity Questionnaire. The specific cut-off values defined by Fried et al. were applied [4].

The Timed Up and Go (TUG) test was also performed: participants were instructed to stand up from a seated position, walk 3 m at a comfortable and safe pace, turn around, walk back to the chair, and sit down again. The time required to complete the task was recorded in seconds.

The Charlson Comorbidity Index (CCI) was calculated based on 16 predefined diseases, without taking additional points for age into account. The following diseases were included in the CCI: myocardial infarction, congestive heart failure (CHF), peripheral vascular disease, cerebrovascular accident (including transient ischemic attacks), dementia, chronic pulmonary disease, connective tissue disease, peptic ulcer disease, liver disease, diabetes mellitus, hemiplegia, moderate to severe chronic kidney disease, solid tumor, leukemia, lymphoma, and AIDS.

Activities of Daily Living (ADL) were assessed using the Barthel Index, which evaluates performance in ten basic self-care activities such as feeding, bathing, grooming, dressing, bowel and bladder control, toilet use, transfers, mobility, and stair climbing [18, 19]. The total score ranges from 0 to 100 points, with higher scores indicating greater independence [18, 19].

Instrumental Activities of Daily Living (IADL) were measured using the Lawton IADL Scale, which assesses more complex activities necessary for independent living, including using the telephone, shopping, food preparation, housekeeping, laundry, transportation, medication management, and handling finances [20]. The total score ranges from 0 to 8 points, with a higher score reflecting greater functional independence [20].

Nutritional status was assessed using the Mini Nutritional Assessment – Short Form (MNA-SF), a validated screening tool for malnutrition in older adults. The MNA-SF includes six questions covering food intake, weight loss, mobility, psychological stress or acute illness, neuropsychological problems, and body mass index (BMI) [21]. The total score ranges from 0 to 14 points, with higher scores demonstrating better nutritional status [21].

The cognitive assessment was performed using the Mini-Mental State Examination (MMSE). For this study, we used the German version of the MMSE, which is incorporated within the licensed software ID DIACOS. The software license includes the permission to use the MMSE scale. The MMSE assesses orientation, registration, attention and calculation, recall, language, and visuoconstructional skills. Scores range from 0 to 30, with higher scores representing better cognitive performance [22].

### Frailty assessment tools

The five items of the SFS were completed as a self-report questionnaire in accordance with the description by Bahat et al. [16]. The cut-off value for classification as frail was set at a score of 3 or more points in accordance with the original protocol [16].

The FFP was used as the reference standard, as described above. The five components of the FFP were recorded according to the procedure used in the original Cardiovascular Health Study [4]. The same thresholds (frail ≥ 3) were used as in the original publication [4].

## Results

### Phase 1: translation, cultural adaptation and reliability testing

According to the protocol, the first five steps of the translation process were completed and approved by Gulistan Bahat. In Step Six, 10 participants (five men and five women) tested the pre-final version. No further cultural adaptations were necessary, and the final version was subsequently approved. The German version of the SFS can be found as Supplementary Table 1.

The last two steps of the protocol involved testing the inter-rater reliability and the test-retest reliability with 20 participants (10 men and 10 women).

The inter-rater reliability demonstrated an ICC of 0.975 (95% CI: 0.911–0.993). The ICC for the test-retest reliability was 0.808 (95% CI: 0.288–0.948).

### Phase 2: clinical validation

A total of 100 patients were included in the study, comprising 70 from the day clinic and 30 from the acute geriatric ward, with a mean age of 82 ± 6.5 years. Of the participants, 66% were female. Based on the SFS, 50 individuals were classified as frail (SFS ≥ 3), while 50 were considered non-frail (SFS < 3). Using the FFP as reference standard, 48 participants were classified as frail. Frail participants were significantly older (*p* = 0.012) and more frequently required walking aids (*p* < 0.001). A significantly greater proportion of non-frail individuals were treated in the day clinic (*p* = 0.002). Furthermore, frail patients exhibited higher comorbidity scores (*p* = 0.022) and used a greater number of regularly prescribed medications (*p* = 0.023). All baseline characteristics are displayed in Table 1.

**Table 1:** Patient characteristics

	All	SFS < 3	SFS ≥ 3	*p*-value
	*N* = 100	*N* = 50	*N* = 50	
Anamnestic and demographic				
Female (yes/no, n, %)	66 (66)	35 (70)	31 (62)	0.398
Age (years)	82 (6.5)	80 (6.4)	83 (6.2)	**0.012**
Weight (kg)	68.7 (16.2)	70.3 (17.9)	67.0 (15.1)	0.326
Height (cm)	165.4 (9.3)	165.8 (9.8)	164.9 (8.9)	0.624
BMI (kg/m²)	25.0 (5.3)	25.4 (5.5)	24.6 (5.1)	0.438
Education level (n, %) no qualification primary school secondary school college	6 (6) 37 (37) 33 (33) 24 (24)	2 (4) 18 (36) 18 (36) 12 (24)	4 (8) 19 (38) 15 (30) 12 (24)	0.809
Living alone (yes/no, n, %)	66 (66)	29 (58)	37 (74)	0.091
Need for walking aid (yes/no, n, %)	43 (43)	9 (18)	34 (68)	**< 0.001**
*Clinical data*				
Day Clinic Patient (yes/no, n, %)	70 (70)	42 (84)	28 (56)	**0.002**
CCI (total score)	2.0 (2.4)	1.5 (2.2)	2.5 (2.4)	**0.022**
Number of regularly used medications	10 (3.8)	9 (3.8)	11 (3.7)	**0.023**
Ex-smoker (yes/no, n, %)	45 (45)	22 (44)	23 (46)	0.841
Current smoker (yes/no, n, %)	4 (4)	2 (4)	2 (4)	1.000
Current alcohol consumption (yes/no, n, %)	47 (47)	26 (52)	21 (42)	0.316
Alcohol intake (glasses/week)	4 (3.3)	4 (2.7)	5 (4.0)	0.465
Falls in the past 12 months (yes/no, n, %)	48 (48)	19 (38)	29 (58)	0.054
*Functionality and muscle strength*				
ADL (Barthel Index, total score)	79 (25.8)	89 (18.4)	68 (27.8)	**< 0.001**
IADL (Lawton Index, total score)	7 (1.6)	8 (1.1)	6 (1.7)	**< 0.001**
Handgrip strength (kg)	19 (8.4)	22 (9.2)	16 (6.3)	**< 0.001**
Timed Up and Go Test (s)	17.6 (12.2)	11.9 (5.8)	24.0 (14.2)	**< 0.001**
4.57-meter gait speed test (s)	7.4 (4.2)	4.9 (1.8)	9.8 (4.4)	**< 0.001**
Gait speed (m/s)	0.8 (0.4)	1.0 (0.5)	0.5 (0.2)	**< 0.001**
Activities per week (Minnesota Leisure Activity questionnaire, kcal)	3070 (4089)	4020 (3663)	2120 (4305)	**0.019**
*Nutritional status*				
MNA-SF (total score)	10 (3.0)	11 (2.8)	9 (3.0)	**< 0.001**
Unintentional weight loss (yes/no, n, %)	47 (47)	18 (36)	29 (58)	**0.028**
Weight loss in total (kg)	7.0 (7.1)	4.8 (3.0)	8.2 (8.4)	0.108
*Cognitive status*				
MMSE (total score)	27 (2.9)	27 (3.0)	26 (2.8)	**0.046**

Table 1: *BMI* Body mass index, *CCI* Charlson Comorbidity Index, *ADL* Activities of Daily Living, *IADL* Instrumental Activities of Daily Living, *kcal* kilocalories, *MNA-SF* Mini Nutritional Assessment – Short Form, *MMSE* Mini-Mental State Examination

Data presented as mean ± Standard deviation if not differently indicated, p-value <0.05 are indicated in bold

Table 2 presents the mean scores (± SD) of the SFS total score and its five components for the overall sample (*N* = 100), as well as for non-frail (SFS < 3; *N* = 50) and frail individuals (SFS ≥ 3; *N* = 50). The mean total SFS score was significantly higher in the frail group (3.52 ± 1.22) compared to the non-frail group (1.92 ± 1.24; *p* < 0.001). Significant group differences were observed across most individual components, including weight loss, fatigue/exhaustion, physical activity, and gait speed (p-values < 0.001). Only the SFS handgrip strength item did not differ significantly between groups (Table 2, *p* = 0.093), whereas objectively measured handgrip strength showed a significant difference (Table 1, *p* < 0.001).

**Table 2:** Results of SFS for frail (SFS ≥ 3) and control (SFS < 3)

Mean (SD)	All	SFS < 3	SFS ≥ 3	*p*-value
	*N* = 100	*N* = 50	*N* = 50	
Total Score (0–5 points)	2.72 (1.46)	1.92 (1.24)	3.52 (1.22)	**< 0.001**
Q1 Weight loss (0–1 point)	0.26 (0.44)	0.08 (0.27)	0.44 (0.50)	**< 0.001**
Q2 Handgrip strength (0–1 point)	0.66 (0.48)	0.58 (0.50)	0.74 (0.44)	0.093
Q3a + b Fatigue/Exhaustion (0–1 point)	0.48 (0.50)	0.26 (0.44)	0.70 (0.46)	**< 0.001**
Q4 Physical activity (0–1 points)	0.62 (0.49)	0.44 (0.50)	0.80 (0.40)	**0.002**
Q5 Gait speed (0–1 points)	0.70 (0.46)	0.56 (0.50)	0.84 (0.37)	**0.002**

*Q* Question, *SFS* Simpler Modified Fried Frailty Scale

Data presented as mean ± Standard deviation, p-value <0.05 are indicated in bold

#### Psychometric properties of the German version of the SFS

### Discriminative power

The SFS total score and all individual items showed strong discriminative ability in identifying frail individuals (FFP ≥ 3) compared to controls (FFP < 3), after adjusting for age and sex. The total SFS score was significantly associated with frailty, with an odds ratio (OR) of 2.954 (95% CI: 1.877–4.649; *p* < 0.001), indicating that each one-point increase nearly tripled the odds of being classified as frail.

Among the individual components, unintentional weight loss (Q1) had the highest discriminative power (OR = 7.316, 95% CI: 2.377–22.517; *p* < 0.001), followed by fatigue/exhaustion (Q3a + b) (OR = 6.579, 95% CI: 2.669–16.217; *p* < 0.001), low physical activity (Q4) (OR = 6.320, 95% CI: 2.408–16.587; *p* < 0.001), and slow gait speed (Q5) (OR = 4.000, 95% CI: 1.490–10.733; *p* = 0.006).

Although its discriminative power was lower than that of the other domains, reduced handgrip strength (Q2) also shown a statistically significant link with frailty (OR = 2.564, 95% CI: 1.041–6.312; *p* = 0.041).

#### Discriminative power

The SFS total score and all individual items showed strong discriminative ability in identifying frail individuals (FFP ≥ 3) compared to controls (FFP < 3), after adjusting for age and sex. The total SFS score was significantly associated with frailty, with an odds ratio (OR) of 2.954 (95% CI: 1.877–4.649; *p* < 0.001), indicating that each one-point increase nearly tripled the odds of being classified as frail.

Among the individual components, unintentional weight loss (Q1) had the highest discriminative power (OR = 7.316, 95% CI: 2.377–22.517; *p* < 0.001), followed by fatigue/exhaustion (Q3a + b) (OR = 6.579, 95% CI: 2.669–16.217; *p* < 0.001), low physical activity (Q4) (OR = 6.320, 95% CI: 2.408–16.587; *p* < 0.001), and slow gait speed (Q5) (OR = 4.000, 95% CI: 1.490–10.733; *p* = 0.006).

Although its discriminative power was lower than that of the other domains, reduced handgrip strength (Q2) also shown a statistically significant link with frailty (OR = 2.564, 95% CI: 1.041–6.312; *p* = 0.041).

### Internal consistency

The internal consistency of the SFS, assessed using Cronbach’s Alpha, was 0.692. All five domains included in the SFS showed a statistically significant and positive correlation with the total score. Correlation coefficients ranged from *r* = 0.533 (handgrip strength) to *r* = 0.685 (physical activity), with all p-values < 0.001 (Table 3).

**Table 3:** Internal consistency by results of the correlation between each domain and the total score of the SFS

Correlation (*n* = 100)			
		*r*	*p*-value
Q1 Weight loss		0.566	**< 0.001**
Q2 Handgrip strength		0.533	**< 0.001**
Q3a + b Fatigue/Exhaustion		0.630	**< 0.001**
Q4 Physical activity		0.685	**< 0.001**
Q5 Gait speed		0.663	**< 0.001**

*Q* Question, *SFS* Simpler Modified Fried Frailty Scale

Spearman’s correlation. p-value <0.05 are indicated in bold

### Construct validity

Construct validity of the SFS was supported by a strong agreement with the FFP, as reflected by a Cohen’s kappa of 0.80 (*p* < 0.001). Furthermore, all individual domains of the SFS showed significant positive correlations with their corresponding domains of the FFP (Table 4). The strongest correlation was observed for weight loss (*r* = 0.696, *p* < 0.001), followed by fatigue/exhaustion (*r* = 0.525, *p* < 0.001), and physical activity (*r* = 0.300, *p* < 0.001). Handgrip strength (*r* = 0.241, *p* = 0.015) and gait speed (*r* = 0.198, *p* = 0.042) were also significantly correlated, though to a lesser extent (Table 4). Additionally, the correlation between the total scores of SFS and FFP was significant (*r* = 0.205, *p* < 0.001), further supporting the construct validity of the SFS (Table 4).

**Table 4:** Construct validity for frailty by correlation of the individual domains of SFS and the appropriate domains of FFP

Correlation (*n* = 100)			
		*r*	*p*-value
FFP Weight loss		0.696	**< 0.001**
FFP Handgrip strength		0.241	**0.015**
FFP Fatigue/Exhaustion		0.525	**< 0.001**
FFP Physical activity		0.300	**< 0.001**
FFP Gait speed		0.198	**0.042**
FFP total score		0.205	**< 0.001**

Q Question, SFS Simpler Modified Fried Frailty Scale, FFP Fried Frailty Phenotype

Spearman’s correlation. p-value <0.05 are indicated in bold

### Diagnostic accuracy of the SFS compared to the FFP

Based on the 2 × 2 classification of the SFS against the FFP, the SFS demonstrated high diagnostic accuracy. Sensitivity was 91.67% and specificity was 88.46%, indicating that the SFS correctly identified the majority of frail and non-frail individuals (Table 5). The positive predictive value (PPV) was 88.00%, and the negative predictive value (NPV) was 92.00% (Table 5). Furthermore, the positive likelihood ratio (LR⁺) of 7.97 and the negative likelihood ratio (LR⁻) of0.09 suggest that the SFS is a strong tool for ruling in and ruling out frailty when compared to the FFP as reference standard.

**Table 5:** Diagnostic performance of the SFS against the FFP

Metric	Value	95% CI
Sensitivity (%)	91.67	80.02–97.68
Specificity (%)	88.46	76.56–95.65
Positive Predictive Value (PPV) (%)	88.00	77.47–93.99
Negative Predictive Value (NPV) (%)	92.00	81.74–96.73
Positive Likelihood Ratio (LR⁺)	7.97	3.72–16.94
Negative Likelihood Ratio (LR⁻)	0.09	0.04–0.24

*SFS* Simplified Modified Fried Frailty Scale, *FFP* Fried Frailty Phenotype, *CI*  Confidence interval, *PPV*  Positive Predictive Value,* NPV*  Negative Predictive Value, *LR⁺* Positive Likelihood Ratio,* LR⁻* Negative Likelihood Ratio

### ROC analysis of the SFS

**Fig. 1:**
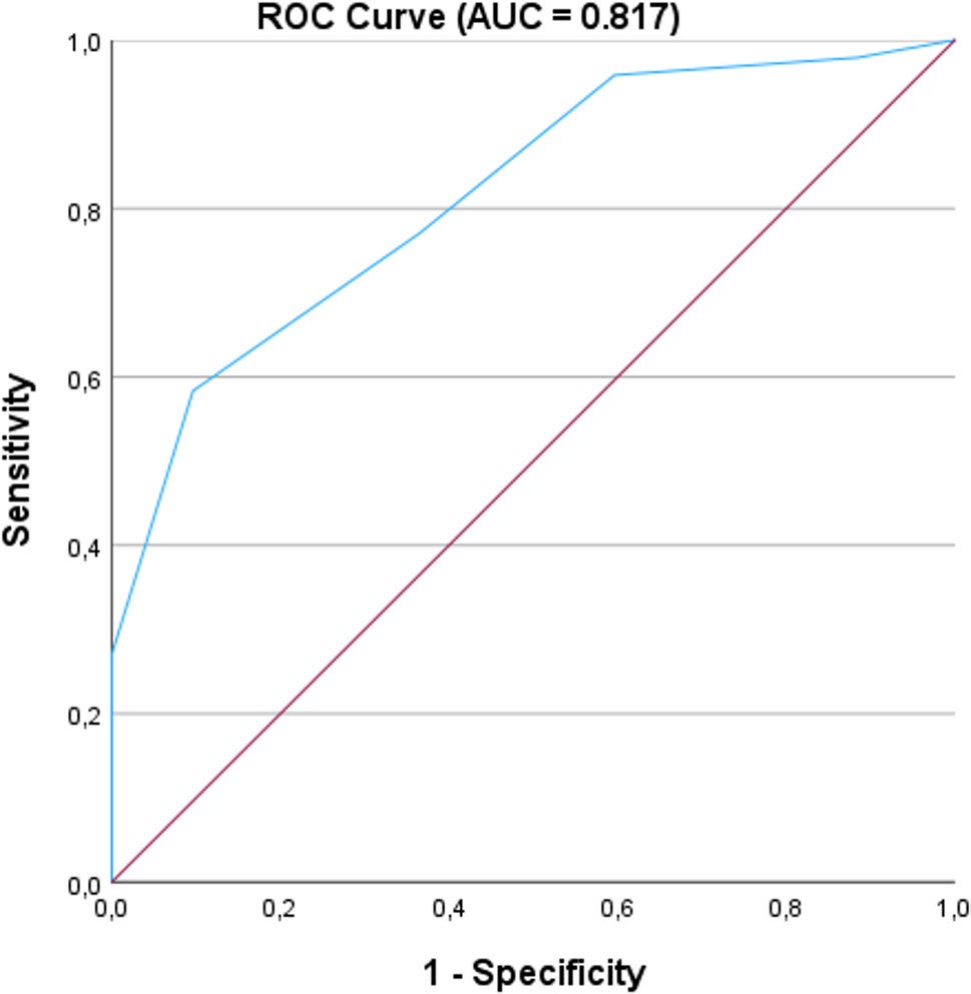
Receiver Operating Characteristic (ROC) curve for the SFS score (0–5 points) in detecting frailty based on the FFP (frail vs. control). The red diagonal represents the line of no discrimination (AUC = 0.5), corresponding to a non-informative classifier

Discriminative ability of the SFS was evaluated using ROC analysis, with frailty status assess by the FFP as the reference standard. The area under the curve (AUC) was 0.817 (95% CI: 0.734–0.899), indicating good accuracy in distinguishing frail from non-frail individuals (Fig. 1).

## Discussion

To the best of our knowledge, this is the first study to validate a German version of the SFS, including its cultural adaptation, demonstrating high reliability and clinically relevant validation results:

### Discriminative validity of the SFS and limitations of self-reported handgrip strength

The findings of our study indicate that the SFS total score, as well its individual components, demonstrates a strong ability to discriminate between frail and non-frail individuals even after adjusting for age and sex (Table 6). There was a significant association between the total score and frailty status, with the likelihood of being frail almost tripling with each additional point (Table 6). This suggests that the cumulative burden of symptoms, as measured by the SFS, is a relevant marker of frailty in older adults.

**Table 6:** Discriminative power of the SFS for frail patients adjusted for age and sex

Frail (*n* = 48, FFP ≥ 3) vs. control (*n* = 52, FFP < 3)			
	OR	95% CI	*p*-value
Total Score	2.954	1.877–4.649	**< 0.001**
Q1 Weight loss	7.316	2.377–22.517	**< 0.001**
Q2 Handgrip strength	2.564	1.041–6.312	**0.041**
Q3a + b Fatigue/Exhaustion	6.579	2.669–16.217	**< 0.001**
Q4 Physical activity	6.320	2.408–16.587	**< 0.001**
Q5 Gait speed	4.000	1.490- 10.733	**0.006**

*Q*  Question, *SFS*  Simpler Modified Fried Frailty Scale, *OR*  odds ratio, *CI*  confidence interval

p-value <0.05 are indicated in bold

Among the individual components, unintentional weight loss, fatigue/exhaustion, low physical activity, and reduced gait speed were all significantly associated with frailty, with odds ratios ranging from approximately 4.0 to 7.3 (Table 6). These components are in line with established markers of physical frailty and are consistent with previous research highlighting their predictive value for adverse outcomes such as disability, hospitalization, and mortality [23–26].

Although handgrip strength was significantly associated with frailty status (OR = 2.56; *p* = 0.041), its discriminatory power was lower compared to the other SFS components (Table 6) and did not reach statistical significance prior to adjustment for age and sex (Table 2). One possible explanation is that handgrip strength is difficult to assess accurately through a self-reported question, as individuals may have limited awareness or subjective perception of their actual muscle strength. Compared to objective handgrip dynamometry, the questionnaire-based approach may lack precision and underrepresent functional deficits. This interpretation is further supported by baseline comparisons, where objectively measured handgrip strength differed significantly between frail and non-frail individuals (Table 1, *p* < 0.001). Thus, handgrip strength remains a robust marker of frailty when measured directly, but appears to be insufficiently captured by self-report. Despite this limitation, the overall discriminative performance of the SFS remained strong, even after adjustment for age and sex, supporting its clinical utility as a frailty screening tool.

### Internal consistency of the SFS

The internal consistency of the German SFS proved to be acceptable with a Cronbach’s alpha of 0.692. This value is within the range that is considered methodologically appropriate in screening instruments with few dichotomous items. Especially with short scales - such as the SFS with only five items - moderate alpha values are to be expected and are not necessarily an indication of a lack of reliability, but rather an expression of the limited number of items and heterogeneity of the measurement areas [27].

All five items showed a significant and positive correlation with the total score, which indicates that they consistently contribute to the superordinate construct of frailty. The highest correlations were observed for physical activity (*r* = 0.685) and gait speed (*r* = 0.663). These results indicate that functional aspects play a central role in the overall construct of frailty as defined by the SFS.

### Strong agreement of SFS with FFP

The results of the construct validity and ROC analysis clearly support the diagnostic quality of the German version of the SFS. The agreement with the reference standard FFP, measured using Cohen’s Kappa of 0.80, is classified as substantial agreement according to commonly used interpretation guidelines, which define values between 0.61 and 0.80 as substantial and 0.81 to 1.00 as almost perfect agreement. Notably, the observed value lies at the upper end of the substantial range, indicating that the SFS demonstrates a high level of consistency with the established FFP.

In addition, the AUC of 0.82 (95% CI: 0.73–0.90) from the ROC analysis demonstrates good discriminatory power between frail and non-frail individuals. An AUC value above 0.8 is generally considered good test quality. This means that the SFS is an effective tool for identifying frailty and can reliably differentiate between affected and non-affected individuals. These results support the clinical applicability of the SFS as a practical and valid alternative to the FFP, especially in time- and resource-limited care settings.

### Comparison to other frailty assessment tools

In addition to the SFS and the FFP, there are numerous other questionnaires for evaluating frailty. A systematic review by Faller et al. from 2019 identified 51 different instruments for frailty evaluation with a range of 3 to 92 items [28]. However, the majority of these questionnaires lack comprehensive validation [28]. Four questionnaires - FRAGIRE, FRAIL Scale, Edmonton Frailty Scale and IVCF-20- have been most frequently examined in terms of their clinimetric properties [28].

First of all, the FRAGIRE (Frailty Groupe Iso-Ressource Evaluation) questionnaire consists of 19 items and was validated in France by Vernerey et al. [29]. It demonstrated an internal consistency (Cronbach’s alpha) of 0.69 and an AUC of 0.85—metrics that are comparable to those of the SFS, despite its more extensive item set [29]. Secondly, FRAIL Scale (Fatigue, Resistance, Aerobic, Illness and Loss of weight), a five-domain instrument developed by the International Association of Nutrition and Aging., was also tested against the FFP and showed a specificity of 86%, but only a sensitivity of 63.6% and an AUC of 0.75 [30, 31]. These values suggest a weaker diagnostic performance compared to the SFS. Thirdly, the Edmonton Frailty Scale from Canada comprises 11 items and achieved a Cronbach’s alpha of 0.62 [32]. In the Australian adaptation for use in acute inpatient geriatric patients, this value was 0.68 [33]. Finally, the IVCF-20 (Clinical-Functional Vulnerability Index), which covers eight domains, stands out for its particularly good test quality criteria [34]. The internal consistency was 0.861 in Reference Center for Older Adults and 0.740 in community dwelling older adults [34]. The best AUC of 0.903 was reached in the reference center population [34].

In summary, only the IVCF-20 outperformed the SFS. All other instruments performed equally or worse, despite some having a higher number of items. This illustrates how difficult it is to measure frailty as a syndrome and underlines the high quality of the test quality criteria of the validated SFS in direct comparison.

### Comparison to the Spanish SFS

A Spanish validation of the SFS was published in May 2025, reporting excellent inter-rater and test-retest reliability (kappa = 0.83 and 0.86, respectively), but only moderate internal consistency (Cronbach’s alpha = 0.67) and criterion validity (Cohen’s kappa = 0.50–0.51 when compared with the modified Fried and FRAIL scales). Sensitivity was limited at 63%, although specificity remained high at 89% [35]. By contrast, the German version of the SFS demonstrated consistently stronger psychometric properties across all domains.

### Strength and limitations

One of the strengths of this study is the systematic translation and cultural adaptation of the SFS, conducted in accordance with a published methodological paper [16] and in consultation with the original developers of the scale. The psychometric properties of the German version were thoroughly evaluated, including both reliability and validity. The inclusion of patients from two distinct geriatric care settings (day clinic and acute ward) ensured a heterogeneous sample, thereby enhancing the clinical applicability and relevance of the findings.

However, this study also has certain limitations. Recruitment was limited to specialized geriatric institutions, which may reduce the generalizability of the findings to other healthcare settings, such as primary care. In addition, individuals who were unable to perform standardized handgrip strength testing were excluded in accordance with the validation protocol, which may have led to an underrepresentation of the most severely impaired individuals and could have contributed to the lower discriminative power observed for this domain. Moreover, the cross-sectional nature of the study precludes any conclusions about the predictive validity of the German SMFFS—namely, its capacity to forecast adverse health outcomes linked to frailty. However, evidence of such predictive validity has been reported for the Turkish version of the scale [9, 17].

## Conclusion

This study presents the validation of the German version of the SFS. The results demonstrate that the SFS is a valid tool for assessing frailty in older adults. It shows substantial agreement with the FFP, has good diagnostic properties, and can be implemented in clinical practice with minimal time and resource requirements. Further validations of the SFS in Turkey and Poland are forthcoming. The German version of the SFS therefore provides a solid foundation for systematic frailty screening in German-speaking countries.

## Supplementary Material

Supplementary Material 1.

## Data Availability

The datasets used and analysed during the current study is available from the corresponding author on reasonable request.

## Abbreviations

ADL: Activities of Daily Living
AUC: Area Under the Curve
BMI: Body Mass Index
CCI: Charlson Comorbidity Index
CI: Confidence Interval
FFP: Fried Frailty Phenotype
FRAIL: Fatigue, Resistance, Aerobic, Illness, and Loss of weight
FRAGIRE: Frailty Groupe Iso-Ressource Evaluation
ICC: Intraclass Correlation Coefficient
IADL: Instrumental Activities of Daily Living
IVCF-20: Clinical-Functional Vulnerability Index (Índice de Vulnerabilidade Clínica Funcional)
LMU: Ludwig-Maximilian-University
LR⁺: Positive Likelihood Ratio
LR⁻: Negative Likelihood Ratio
MMSE: Mini-Mental State Examination
MNA-SF: Mini Nutritional Assessment – Short Form
NPV: Negative Predictive Value
OR: Odds Ratio
PPV: Positive Predictive Value
ROC: Receiver Operating Characteristic
SD: Standard Deviation
SFS: Simpler Modified Fried Frailty Scale
TUG: Timed Up and Go
