# Supplementary material for: The German simpler modified fried frailty scale: translation, cross-cultural adaptation and clinical validation
Source: BMC Geriatr. 2026 Mar 5;26:482. doi: 10.1186/s12877-026-07134-1 (PMC13064104; doi:10.1186/s12877-026-07134-1)

**Supplementary Part:**

**Supplementary Table 1:**

**German version of the Simpler Modified Fried Frailty Phenotype (SFS):**


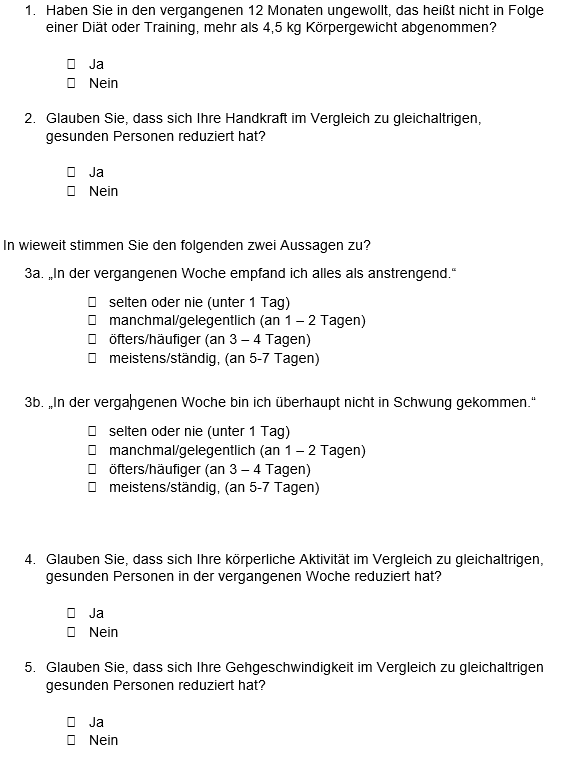

Supplement: Supplementary file 1 — Supplementary Material 1. [file 12877_2026_7134_MOESM1_ESM.docx]
